# Cranial Osteology of Meiglyptini (Aves: *Piciformes*: *Picidae*)

**DOI:** 10.1155/2012/951836

**Published:** 2012-01-22

**Authors:** Reginaldo José Donatelli

**Affiliations:** Laboratory of Ornithology, Department of Biological Sciences, Sciences College, São Paulo State University (UNESP), Caixa Postal 473, 17001-970 Bauru, SP, Brazil

## Abstract

The Meiglyptini comprise eight species grouped into three genera: *Meiglyptes* and *Mulleripicus*, with three species each, and *Hemicircus,* with two species. The aim of the present study was to describe the cranial osteology of six species and three genera of Meiglyptini and to compare them to each other, as well as with other species of woodpeckers and other bird groups. The cranial osteology varied among the investigated species, but the most markedly distinct characteristics were: (1) a frontal overhang is only observed in the middle portion of the frontale of *H. concretus*; (2) the Proc. zygomaticus and suprameaticus are thick and long in species of the genus *Mulleripicus*, but short in other species; (3) the *Pes pterygoidei* is relatively larger in species of the genus *Mulleripicus*, while it is narrow, thin and relatively smaller in species of the genus *Meiglyptes* and indistinct in *H. concretus*; (4) the bony projection of the ectethmoidale is relatively short and thin in species of *Mulleripicus* and more developed in *H. concretus*. It appears that the greatest structural complexity of the cranial osteology is associated with the birds' diet, with the frugivorous *H. concretus* being markedly different from the insectivorous species.

## 1. Introduction

Woodpeckers have been investigated scientifically for over a century. These birds are notable for their colour, size, foraging mode, nest-building behaviour, instrumental signals, and the way they climb vertical surfaces. Interestingly, many of the behavioural patterns of woodpeckers are closely related to their anatomical features. Thus, these aspects cannot be dissociated in a study [1].

The Meiglyptini comprise three genera of Old World woodpeckers: *Meiglyptes *and *Mulleripicus*, with three species each, and *Hemicircus*, with two species [2]. These species are all arboreal and feed mainly upon the larvae and eggs of insects, ants and secondarily termites, beetles, caterpillars, and other arthropods. There is also a frugivorous species in this group, *H. concretus*.

The foraging modes vary among these woodpeckers, irrespective of food type. The Meiglyptini primarily employ gleaning, with probing and tapping being used secondarily; excavating and tonguing are less common.

The aim of the present study was to describe and compare the mandibular apparatus of several species of *Meiglyptini* and perform a morphofunctional analysis of the complexity of this apparatus, relating it with the species' foraging mode. Specifically, the following questions were addressed:

 Are the foraging mode and the structure of the mandibular apparatus related among the Meiglyptini?; and is it possible to establish any relationship between form and function based on structural differences between the jaw apparatus and the foraging modes? Thus, there are at least two hypotheses to be considered: (1) there is no relationship between the structural complexity of the mandibular apparatus in Meiglyptini and the foraging mode, and (2) there is a relationship between the structure of the mandibular apparatus and the foraging mode in Meiglyptini.

## 2. Materials

In this study, I described the osteological characters of the skull of 15 specimens of Meiglyptini belonging to three genera and six species. The specimens are housed at the National Museum of Natural History (USNM), Smithsonian Institution, Washington DC, USA, the Museum Zoologicum Bogoriense (MZB), Indonesian Institute of Sciences (LIPI), and the Natural History Museum of the Indonesian Institute of Sciences, Indonesia.

Bellow is a list of the species investigated after Winkler and Christie [2], museum abbreviations and the relative numbers of specimens in the collections. All specimens preserved in 70% ethanol were from the Museum Zoologicum Bogoriense, Indonesian Institute of Sciences (LIPI), whereas the others were osteological material: *Hemicircus concretus *(Temminck, 1821) LIPI MZB.Skt 125 Hc1; LIPI MZB.SKT 126 HC2; *Meiglyptes tristis *(Horsfield, 1821) LIPI MZB.Skt Mtr1 123; MZB.Skt Mtr2 124; USNM 292,228 ♂; *Meiglyptes tukki *(Lesson, 1839) LIPI MZB.Skt Mtu1 121; MZB.Skt Mtk1 122; USNM 489,269 ♀; *Mulleripicus pulverulentus *(Temminck, 1826) LIPI MZB.Skt Mp1 127; MZB.Skt 128 Mp2; ♀ USNM 19201, USNM 562,042 ♀; *Mulleripicus fulvus *(Quoy and Gaimard, 1830) USNM 491,227 ♀, USNM 226,191 ♀; *Mulleripicus funebris *(Valenciennes, 1826) ♂ USNM 489,265.

## 3. Methods

The osteology of the skull and mandible was studied comparatively, described, and drawn using a Zeiss Stemi SV11 stereomicroscope (http://www.zeiss.com/) with magnification ranging from 4 to 66X. *M. pulverulentus *was used as a reference for comparison of structures. All drawings are accompanied by legends to facilitate the observation of structures.

The nomenclature used to describe the cranial osteology follows the Nomina Anatomica Avium (NAA; [3, 4]). When no reference to a particular structure was available, I used letters and numbers to avoid the unnecessary creation of names. The species nomenclature follows Winkler and Christie [2].

## 4. Results

### 4.1. Osteology

**Ossa cranii**

**Figure 1:**
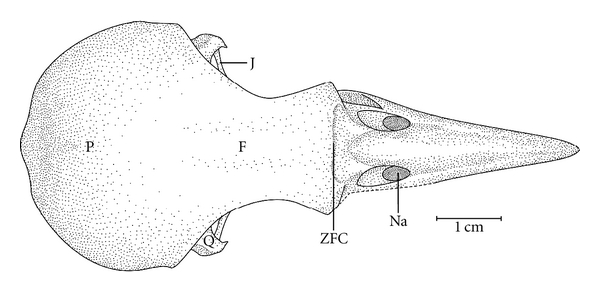
Dorsal view of the skull of *Meiglyptes tristis*. F: frontal region; J: jugal arch; NA: nostril; P: parietal region; Q: quadrate bone; ZFC: craniofacial flexion zone.

**Figure 2:**
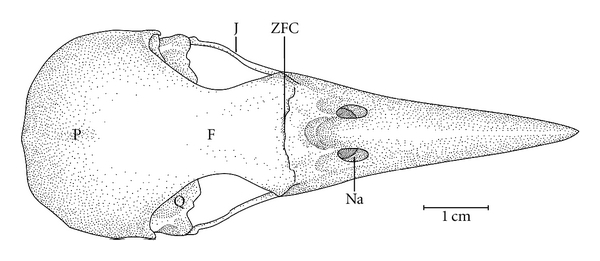
Dorsal view of the skull of *Meiglyptes tukki*. F: frontal region; J: jugal arch; NA: nostril; P: parietal region; Q: quadrate bone; ZFC: craniofacial flexion zone.

**Figure 3:**
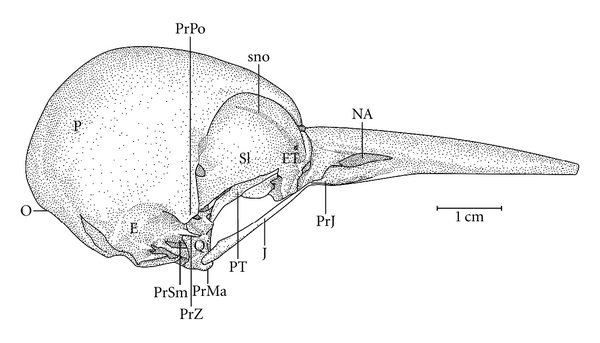
Lateral view of the skull of *Hemicircus concretus*. E: squamosal region; ET: ectethmoid region; J: jugal arch; NA: nostril; P: parietal region; PjJ: jugal projection; PrMA: mandibular process of the quadrate bone; PrZ: zygomatic process; PrPO: postorbital process; PrSm: suprameatic process; PT: pterygoid bone; Q: quadrate bone; SI: interorbital septum; sno: olfactory ridge; O: occiptal region.

**Figure 4:**
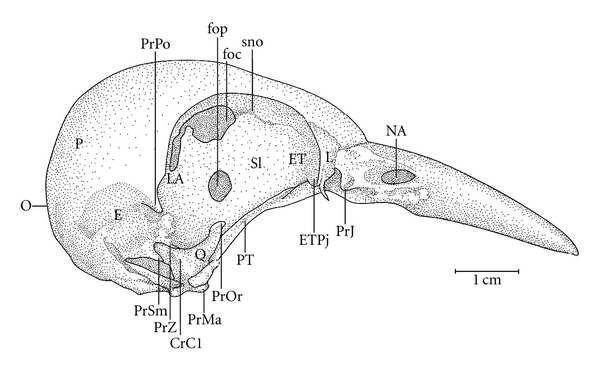
Lateral view of the skull of *Meiglyptes tristis*. CrC1: C1 crest; E: squamosal region; ET: ectethmoid region; ETPj: ethmoid bony projection; foc: foramen; fop: optical foramen; J: jugal arch; L: lacrimal region; LA: laterosphenoid bone; NA: nostril; P: parietal region; PjJ: jugal projection; PrMa: mandibular process of the quadrate bone; PrOr: orbital process of the quadrate bone; PrPO: postorbital process; PrSm: suprameatic process; PrZ: zygomatic process; PT: pterygoid bone; Q: quadrate bone; SI: interorbital septum; sno: olfactory ridge; O: occiptal region.

**Figure 5:**
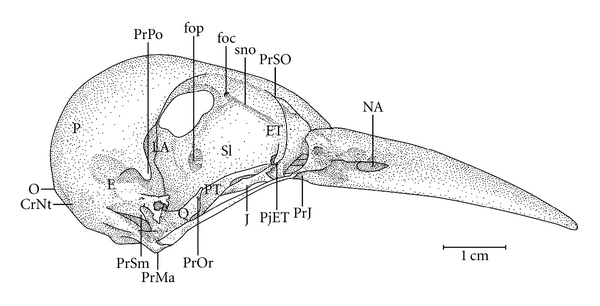
Lateral view of the skull of *Meiglyptes tukki*. CrNt: transverse nuchalis crest; E: squamosal region; ET: ecthetmoid region; PjET: ethmoid bony projection; foc: foramen; fop: optical foramen; J: jugal arch; LA: laterosphenoid bone; NA: nostril; P: parietal region; PjJ: jugal projection; PrMa: mandibular process of the quadrate bone; PrOr: orbital process of the quadrate bone; PrPO: postorbital process; PrSm: suprameatic process; PrZ: zygomatic process; PT: pterygoid bone; Q: quadrate bone; SI: interorbital septum; sno: olfactory ridge; O: occiptal region.

**Figure 6:**
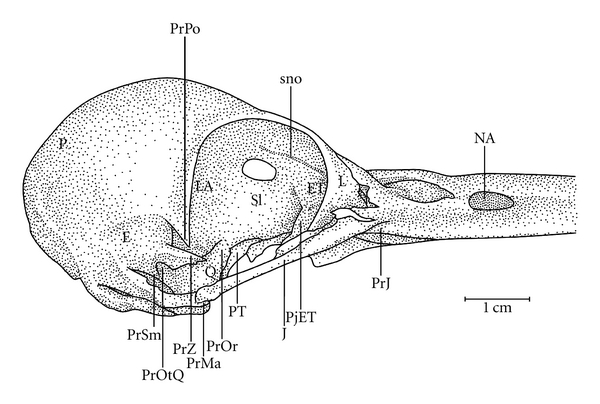
Lateral view of the skull of *Mulleripicus pulverulentus*. E: squamosal region; ET: ectethmoid region; PjET: ethmoid bony projection; J: jugal arch; L: lacrimal region; LA: aterosphenoid bone; NA: nostril; P: parietal region; PjJ: jugal projection; PrMa: mandibular process of the quadrate bone; PrOr: orbital process of the quadrate bone; PrOtQ: otic process of the quadrate bone; PrPO: postorbital process; PrSm: suprameatic process; PrZ: zygomatic process; PT: pterygoid bone; Q: quadrate bone; SI: interorbital septum; sno: olfactory ridge.

The Os frontale (F) articulates rostrally with the Os nasalis through the craniofacial flexion zone (ZFC). This is more evident in *M. tristis *(Figure 1) and *M. tukki *(Figure 2) and less in *H. concretus*, whereas it is indistinguishable in *M. pulverulentus*. *Hemicircus concretus* (Figure 3) bears a unique thin bony elevation (Be) at the middle portion of the Os frontale; there is no such elevation in other species. Laterocaudally, the frontal region is connected with the Proc. *postorbitalis* (PrPO). This process is short, with approximately 1/6 of the length occurring between its origin in the skull and the jugal arch in *M. tristis *(Figure 4), 1/5 in *H. concretus* (Figure 3), 1/4 in *M. tukki* (Figure 5), and 1/3 in *M. pulverulentus *(Figure 6). The sutura *frontolacrimalis* is absent, and the Os lacrimale is fusioned with the frontal region in all species.

**Figure 7:**
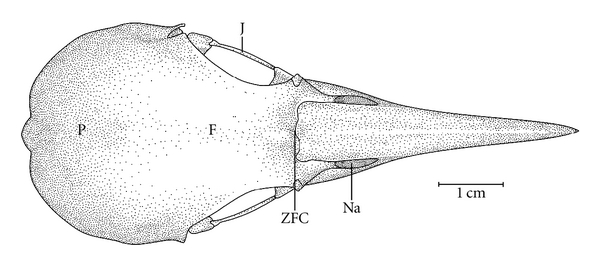
Dorsal view of the skull of *Hemicircus concretus*. F: frontal region; J: jugal arch; NA: nostril; P: parietal region; ZFC: craniofacial flexion zone.

**Figure 8:**
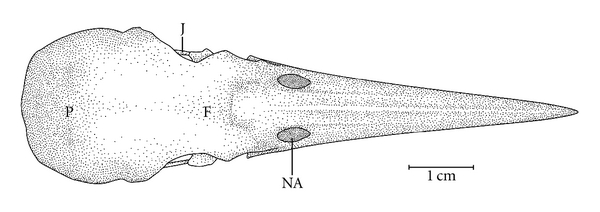
Dorsal view of the skull of *Mulleripicus pulverulentus*. F: frontal region; J: jugal arch; NA: nostril; P: parietal region.

The Os parietale is expanded laterally approximately twice the length of the lateral expansion of the Os frontale in *H. concretus *(Figure 7), about 1.5 times in *M. pulverulentus* (Figure 8), and about 2.5 times in *M. tristis *(Figure 1) and *M. tukki* (Figure 2). 

The values of these parameters indicate not only the frontale/parietale relationship but also provide the skull dimensions in many species. The Os squamosum is connected anteromedially with the Os laterosphenoidale by the crista laterosphenoidale (CrL–Figure 5) and anterocaudally with the Os frontale by the Proc. postorbitalis. The Fossa temporalis is wider than long in all species.

 The Os squamosum is projected rostrally, forming the Proc. zygomaticus (PRZ), which articulates ventrally with the Proc. oticus quadrati and clearly has dorsal, lateral, medial, and ventral faces. This is the region of origin of the aponeurosis of the *M. adductor mandibulae externus ventralis *and the *M. adductor mandibulae externus rostralis lateralis*  (Donatelli, [5]). The Proc. *zygomaticus* (Proc. *squamosal*, [6]) is thick and long in species of the genus *Mulleripicus *(Figure 6) and short in the other species. The Os squamosum also forms the Proc. *suprameaticus* (PrSm–Figure 6), and this is present only in species of the genus *Mulleripicus* (Figure 6).

**Ossa faciei**

The Os *pterygoideum* has a well-developed anterior expansion, known as the *Pes pterygoideus, *which extends rostrally and articulates with the ventral portion of the *septum interorbitale*, the *rostrum parasphenoidale*, and the pars palatina. The *Pes pterygoideus* is observed and well developed in species of *Mulleripicus*, but it is relatively less developed, thin, and narrow in species of *Meiglyptes* and absent in *H. concretus*. Posterodorsally to the Os *pterygoideum* lies the *processus dorsalis*, which includes the aponeurosis of the *M. protractor pterygoidei*. This is only present in species of *Mulleripicus*.

**Figure 9:**
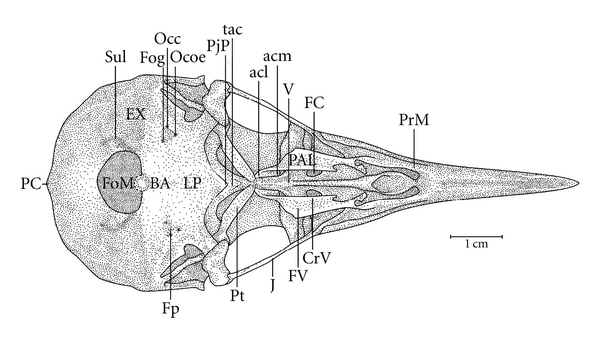
Ventral view of the skull of *Hemicircus concretus*; BA: basioccipital region; CrV: ventral palatine crest; EX: exoccipital region; FC-; Fog-; FoM: foramen magnum; Fp-; FV: ventral fossa; J: jugal arch; LP: lamina *parasphenoidalis*; Occ-; Ocoe-; PAL: palatine; PC-; PjP: projection of the parasphenoid rostrum; PrM: maxilar process of the palatine; PrPA: paraoccipital process; Pt: pterygoid bone; RP: parasphenoidal rostrum; Sul: intercotylar sulcus; V: vomer; Occ: *Ostium canalis carotici*; Ocoe: *Ostium canalis ophthalmici externus*; PC: *Proeminentia cerebellaris*; Fog: *Foramen nervi glossopharyngealis*; FC: *Fossa choanalis*; Fp: *Fossa parabasalis*.

**Figure 10:**
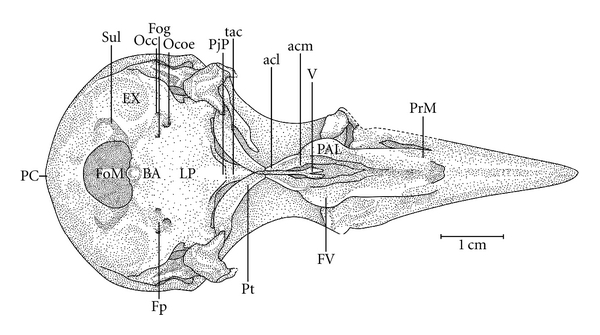
Ventral view of the skull of *Meiglyptes tristis*; BA: basioccipital region; EX: exoccipital region; Fog-; FOM: foramen magnum; Fp-; FV: ventral fossa; LP: lamina *parasphenoidalis*; Occ-; Ocoe-; PAL: palatine; PC-; PjP: projection of the parasphenoid rostrum; PrM: maxilar process of the palatine; Pt: pterygoid bone; Sul: intercotylar sulcus; V: vomer; Occ: *Ostium canalis carotici*; Ocoe: *Ostium canalis ophthalmici externus*; PC: *Proeminentia cerebellaris*; Fog: *Foramen nervi glossopharyngealis*; Fp: *Fossa parabasalis*.

**Figure 11:**
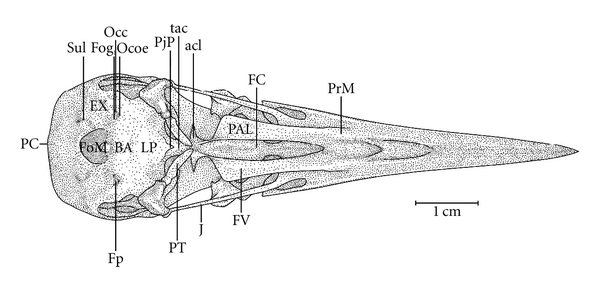
Ventral view of the skull of *Mulleripicus pulverulentus*; BA: basioccipital region; EX: exoccipital region; Fog-; FOM: foramen magnum; FC-; Fp-; FV: ventral fossa; J: jugal arch; LP: lamina *parasphenoidalis*; Occ-; Ocoe-; PAL: palatine; PC-; PjP: projection of the parasphenoid rostrum; PrM: maxilar process of the palatine; PT: pterygoid bone; Sul: intercotylar sulcus; V: vomer; Occ: *Ostium canalis carotici*; Ocoe: *Ostium canalis ophthalmici externus*; PC: *Proeminentia cerebellaris*; Fog: *Foramen nervi glossopharyngealis*; FC: *Fossa choanalis*; Fp: *Fossa parabasalis*.

**Figure 12:**
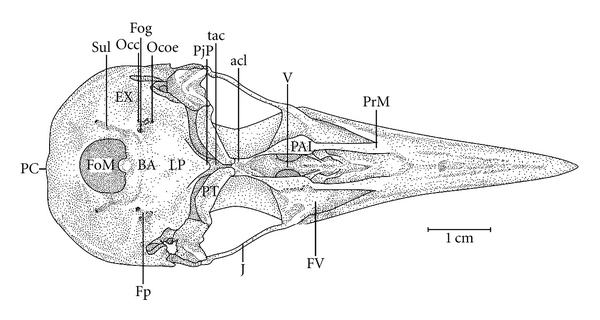
Ventral view of the skull of *Meiglyptes tukki*; BA: basioccipital region; EX: exoccipital region; Fog-; FOM: foramen magnum; FC-; Fp-; FV: ventral fossa; J: jugal arch; LP: lamina *parasphenoidalis*; Occ-; Ocoe-; PAL: palatine; PC-; PjP: projection of the parasphenoid rostrum; PrM: maxilar process of the palatine; PT: pterygoid bone; Sul: intercotylar sulcus; V: vomer; Occ: *Ostium canalis carotici*; Ocoe: *Ostium canalis ophthalmici externus*; PC: *Proeminentia cerebellaris*; Fog: *Foramen nervi glossopharyngealis*; FC: *Fossa choanalis*; Fp: *Fossa parabasalis*.

In the middle portion of the Os *palatinum* lies the *fossa choanalis* (FC), which is delimited by the *crista ventralis* (CrV), forming a gap. This gap varies in size among the species studied. It is relatively wider in *H. concretus* (Figure 9), decreases in width in species of *Meiglyptes *(Figure 10), and is relatively narrow in species of *Mulleripicus* (Figure 11), in which the Os *palatinum* is curved caudally. Between the *cristae ventralis et lateralis* lies the *fossa* ventralis (FV), which is distinct and deep in *M. tristis* (Figure 10), relatively deep and shallow in species of *Mulleripicus* (Figure 11), and shallow in *M. tukki *(Figure 12) and *H. concretus* (Figure 9). This is an important region because it is the origin of the aponeurosis and muscle fibres of the *M. pterygoideus ventralis*  (Donatelli, [5]).

 The Os laterosphenoidale lies caudally to the *facies orbitales*. It is connected laterally with the Os *squamosum* by the crista laterosphenoidale (CrL–Figure 5), where the aponeurosis of the *M. adductor mandibulae externus caudalis medialis *originates  (Donatelli, [5]). This crest is apparent only in species of the genus *Mulleripicus* and in *M. tukki* (Figure 5). It is common to observe a swelling in the connection between the crista laterosphenoidale and the septum interorbitale in all species. The nerve foramen lies ventrolaterally.

The Os ectethmoidale has a bony projection (ETPj–Figures 4–6) reaching the dorsal surface of the *arcus jugalis*, though without fusion. The projection is relatively more apparent in* M. tristis* (Figure 4) than in other species, but it is more developed in *H. concretus* (Figure 3). It assumes a triangular shape in *M. tukki* (Figure 5). In *Mulleripicus* (Figure 6), the projection is relatively short and thin upon the *arcus jugalis*.

The Os quadratum has a *corpus quadrati* that connects with the *proc. oticus quadrati* (PrOtQ), *proc. orbitalis*, and *proc. mandibularis*. The proc. oticus quadrati articulates dorsally with the Os squamosum by the ventral surface of the proc. *zygomaticus* (PRZ), which protrudes dorsally upon the *proc. oticus quadrati* in the species of *Mulleripicus* (Figure 6). This process is relatively shorter in *H. concretus *and *M. tukki*. In species of *Mulleripicus*, there is a greater distance between the *proc*. *orbitalis quadrati* and the posterior portion of the Os laterosphenoidale. In the *proc. oticus quadrati*, a small C1 crest (C1Cr) can be observed in all species but represented in *M. tristis* (Figure 4), which originates from the aponeurosis of the *M. adductor mandibulae externus caudalis lateralis*  (Donatelli, [5]). The *proc. orbitalis quadrati* (PrOr–Figures 4–6) protrudes anteromedially from the *corpus quadrati*. This is a short process, but its form varies greatly among species. In general, it is slender and has a length equivalent to two thirds of the length of the Os *pterygoideus*, which lies medially. The condylus medialis is usually the most developed, compared to other condyles, as observed in most species, and this structure is relatively more developed in *M. tristis*, in which it acquires a protruding and pointed shape. The condylus caudalis is an extension of the condylus lateralis in all species.

The maxilla is formed by the fusion of the Os premaxillare, maxillare, and nasale. It presents approximately half the total length of the skull in most species, except in species of *Mulleripicus* (Figure 8), in which it measures approximately 65% of the total length of the skull.

**Ossa Mandibulae**

The *pars symphisialis mandibulae* (Psi) is short and measures slightly more than one third of the total length of the mandible only in species of *Meiglyptes*. In *M*. *pulverulentus*, the Psi measures approximately 40% of the total length of the mandible, compared to 45% in *H. concretus*.In the dorsal region of the mandible lies the *proc. pseudocoronoideus* 1 (= process of the *M. adductor mandibulae*, [3]). This process is the insertion point of the tendon common to the *M. adductor mandibulae externus rostralis temporalis *and the *M. adductor mandibulae externus rostralis medialis*  (Donatelli, [5]). It is indistinct in all species, except *M. tristis *and species of *Mulleripicus*. Additionally, the proc. *pseudocoronoideus* 2 is relatively indistinct in all species.

On the *pars intermedia*, there is a peculiar depression, the *fossa lateralis mandibulae*, in which the muscle fibres of the *M. adductor mandibulae externus ventralis *are inserted. This region is connected to the posterior portion by the *crista caudalis mandibulae*. This crest reaches the dorsal Proc. *pseudocoronoideus* 2.

In the middle portion of the mandible lies the *proc. medialis mandibulae* (internal jaw process, angular medial process, [6, 7]), which protrudes dorsomedially. This process varies greatly in length in the woodpeckers studied. This is one of the most important structures for the insertion of muscle fibres and their aponeurosis of the complex of the Os *pterygoideum*. The *tuberculum pseudotemporale*, which includes the aponeurosis of the *M. pseudotemporalis superficialis*, is also noticeable. It is a conspicuous structure in most species, except in *H. concretus*. All species bear a shallow *fossa caudalis* in the posterior portion of the mandible. This is the insertion point of the muscle fibres of the *M. depressor mandibulae*.

 Some of the structural differences observed in the components of the cranial osteology of the *Meiglyptini* are noteworthy due to their exclusivity, relative development (being larger or smaller) or unique features present in one group of species within a genus or a species, as follows: (1) there is a thin bony elevation (Be), referred to as the frontal overhang by Bock [8], in the middle portion of the Os frontale in *H. concretus*; (2) the Os parietale is expanded laterally and is equivalent to approximately twice the length of the lateral expansion of the Os frontale in *H. concretus*, 1.5 times in *M. pulverulentus*, and 2.5 times in *M. tristis *and *M. tukki*; (3) the *fossa temporalis* is wider than long in all species; (4) the *proc. zygomaticus* is long and thick in species of the genus *Mulleripicus *and short in the other species; (5) the proc. *suprameaticus* (PrSM) is apparent only in species of the genus *Mulleripicus*; (6) the *Pes pterygoidei* is relatively larger in species of the genus* Mulleripicus*, relatively smaller, thin, and narrow in species of the genus *Meiglyptes*, and indistinct in *H. concretus*; (7) the *fossa choanalis* is relatively wider in *H. concretus* and becomes progressively narrower in species of *Meiglyptes *and *Mulleripicus*; (8) the *fossa ventralis palatina* is deep in *M. tristis* and becomes progressively shallower in *Mulleripicus* species*, M. tukki*, and *H. concretus*; (9) the bony projection of the Os ectethmoidale is relatively short and slender in *Mulleripicus *species and more developed in *H. concretus*; (10) the *condylus medialis* is usually the most developed one in all species, and, in *M. tristis*, it is prominent and pointed; (11) the *pars symphisialis mandibulae* is short and extends for slightly more than 1/3 of total length of the mandible only in species of *Meiglyptes* for approximately 40% in *M. Pulverulentus*, and approximately 45% in *H. concretus*.

## 5. Discussion

This analysis of the cranial osteological structures of the *Meiglyptini* elucidated seven important mechanisms of operation of the jaw apparatus: (1) *Ossa cranii*: frontal overhang, the extension of the Os parietale versus the Os frontale, *fossa temporalis*, *proc*. *suprameaticus*, and *proc*. *zygomaticus*; (2) *Ossa faciei*: anterior expansion of the Os pterygoideum, *Pes pterygoidei*, *fossa choanalis*, *fossa ventralis palatine*, bony projection of the Os ectethmoidale, and *condylus medialis quadrati*; (3) *Ossa mandibulae*: *pars symphisialis mandibulae*. 

*H. concretus *is the only species within the *Meiglyptini* that presents a bony elevation in the Os frontale, which is known as the frontal overhang [8]. Among the species of woodpeckers studied by Donatelli [1], only *Picumnus cirratus *exhibited such a structure though it was later found in species of *Picus*  (Donatelli [5]), and it was described in *Sphyrapicus varius *by Burt [9]. Bock [8] suggested that this structure functions as a “bony stop”, preventing an excessive protraction of the maxilla. This mechanism is more important in woodpeckers specialized to obtain food by drumming (“drilling” or “pecking”) in trees. However, the great majority of the *Meiglyptini* (this study), the Picini  (Donatelli [5]), and the Neo- and Afrotropical woodpeckers [1] that drum on tree bark exhibit no such structure. Moreover, *H. concretus *does not drum on tree trunks. The main foraging strategy of *H. concretus *is gleaning among the canopy; therefore, it is difficult to explain such a complex structure in this species. This structure seems controversial and deserves a broader study of woodpecker species before a more precise conclusion about its function can be drawn.

The variation of the Os parietale in relation to the Os frontale appears to determine the proportions of the skull in many species. However, I found that the smallest species that investigated (*H. concretus*) did not present the largest expansion, similar to what has been found in other studies on woodpeckers ([1], in prep.). The strongest relationship was found in intermediate-sized species of *Meiglyptes* and in species of *Hemicircus *and *Mulleripicus*.

The *proc. zygomaticus* and *suprameaticus*, which are important insertion points of muscles and the aponeurosis of the *M. adductor mandibular *system, were relatively more developed in larger species (*Mulleripicus*) and underdeveloped in other species of *Meiglyptes *and *Hemicircus*. In other groups of woodpeckers, such processes vary in size and shape, and it is not possible to establish a clear relationship between their development and size ([1], in prep.). Jollie [10] suggested that the Os squamosum articulates with the Os quadratum and a short proc. “*zygomaticus*” in chickens, which was also observed in all species of *Picidae* studied here. However, the proper term for this should be *proc. squamosal* and not “*zygomaticus*” [4], as the term “*zygomatic*” is characteristic of mammalian skulls.

The anterior expansion of the Os pterygoideum forms the *Pes pterygoidei*. The length of this process increases in associate with the size of *Meiglyptini* species, from the largest to the smallest in the order *Mulleripicus*, *Meiglyptes*, and *Hemicircus*. This observation diverges from findings of previous studies  (e.g., Donatelli [1, 5]) that found that this structure is well developed and is a unique feature of all woodpeckers. Such a structure was not described in other groups of birds related to the *Piciformes*, such as the *Galbulidae* [11] or *Coraciiformes* [12]. In these birds, the *Pes pterygoidei* is the insertion point of the fibres of the *M. pterygoideus dorsalis medialis*, which is an important muscle retractor of the upper jaw. Burton [7] described the *Pes pterygoidei* in the *Picidae*, *Picumninae*, and *Indicatoridae*. The *proc. pterygoideus dorsalis* [13] is the insertion point of the aponeurosis of the *M. protractor pterygoideus*. This process is conspicuous only in *Mulleripicus* among the *Meiglyptini*. In the *Picidae*  (Donatelli [5]), this process is larger in *B. rubiginosus *and smaller in other species, but always distinct. This process was not mentioned by Burton [7], perhaps because he considered it as only one muscle of the *M. protractor quadrati *system: the *M. protractor pterygoidei et quadrati *(with insertion in the dorsal portion of the articulation of the *pterygoideum-quadratum*).  Donatelli [1, 5] described this muscle as two distinct muscles (*M. protractor pterygoidei *and *M. protractor quadrati*) because they had different origins and insertions. Bock ([14], p. 12) previously called attention to this structure, particularly in woodpeckers.

The *M. pterygoideus ventralis medialis*, which lies in the *fossa ventralis* palatine, is very well developed in woodpeckers and represents a powerful retractor of the upper jaw in birds. Gennip [15] is one of the few authors who described this fossa and related it to the origin and development of the *M. pterygoideus ventralis medialis*. Other authors studying the Columbidae [16, 17] did not mention this structure and only related the development of these muscles in species within the family. According to Bock [14], “the mass and shape of the palate are correlated with the size and power of the upper jaw and with the strength of the muscles. Many of the exact details of this correlation must still be ascertained.” According to Morioka [18], the deeper the *fossa ventralis palatina*, the greater the development of the related muscle mass and the greater the power of retraction of the upper jaw. However, he noted that the poorly developed muscle mass in the Apodidae allowed them to close their beak more rapidly, at the expense of a “powerful biting force.” Among the woodpeckers studied, this structure is relatively deep and conspicuous only in *M. tristis*, whereas the largest size and structural development of the *M. pterygoideus ventralis medialis et lateralis* were found in *M. pulverulentus*.The size and shape of the *proc. orbitalis quadrati* is prominent in *Mulleripicus *species relative to other woodpeckers.The associated *M. pseudotemporalis profundus *is also relatively more developed.  Donatelli [5] reported that* B. rubiginosus *had the largest process among the Picini, and the associated muscle was relatively less developed than in other species. As pointed out by Bock [14], the *condylus medialis mandibulae* is the most developed among the condyles of the Os quadratum. This is similar to what was found in the *Meiglyptini*, especially in *M. tristis*, in which the condyle was even more distinguished in shape. 

We found clear structural differences in the cranial osteology between the frugivorous *H. concretus* and other insectivorous species of *Meiglyptini*. Therefore, natural selection appears to have shaped the jaw apparatus as a whole differently in species with different food types feeding locations, irrespective of their foraging mode. This becomes clear when the development of these structures is considered in species of *Meiglyptes *and *Mulleripicus *compared to *Hemicircus concretus*. These aspects will be further discussed elsewhere based on the results of this study.
